# Deep sequencing of the mouse lung transcriptome reveals distinct long non-coding RNAs expression associated with the high virulence of H5N1 avian influenza virus in mice

**DOI:** 10.1080/21505594.2018.1475795

**Published:** 2018-07-27

**Authors:** Jiao Hu, Zenglei Hu, Xiaoquan Wang, Min Gu, Zhao Gao, Yanyan Liang, Chunxi Ma, Xiaowen Liu, Shunlin Hu, Sujuan Chen, Daxing Peng, Xinan Jiao, Xiufan Liu

**Affiliations:** a Animal Infectious Disease Laboratory, School of Veterinary Medicine, Yangzhou University, Yangzhou, Jiangsu, China; b Jiangsu Co-innovation Center for Prevention and Control of Important Animal Infectious Diseases and Zoonosis, Yangzhou University, Yangzhou, Jiangsu, China; c Jiangsu Key Laboratory of Zoonosis, Yangzhou University, Yangzhou, China; d Key Laboratory of Prevention and Control of Biological Hazard Factors (Animal Origin) for Agri-food Safety and Quality, Ministry of Agriculture of China (26116120), Yangzhou University, Yangzhou, China

**Keywords:** H5N1 influenza virus, deep sequencing, lncRNAs, innate immune response, pathogenesis

## Abstract

Long non-coding RNAs (lncRNAs) play multiple key regulatory roles in various biological processes. However, their function in influenza A virus (IAV) pathogenicity remains largely unexplored. Here, using next generation sequencing, we systemically compared the whole-transcriptome response of the mouse lung infected with either the highly pathogenic (A/Chicken/Jiangsu/k0402/2010, CK10) or the nonpathogenic (A/Goose/Jiangsu/k0403/2010, GS10) H5N1 virus. A total of 126 significantly differentially expressed (SDE) lncRNAs from three replicates were identified to be associated with the high virulence of CK10, whereas 94 SDE lncRNAs were related with GS10. Functional category analysis suggested that the SDE lncRNAs-coexpressed mRNAs regulated by CK10 were highly related with aberrant and uncontrolled inflammatory responses. Further canonical pathway analysis also confirmed that these targets were highly enriched for inflammatory-related pathways. Moreover, 9 lncRNAs and 17 lncRNAs-coexpressed mRNAs associated with a large number of targeted genes were successfully verified by qRT-PCR. One targeted lncRNA (NONMMUT011061) that was markedly activated and correlated with a great number of mRNAs was selected for further in-depth analysis, including predication of transcription factors, potential interacting proteins, genomic location, coding ability and construction of the secondary structure. More importantly, NONMMUT011061 was also distinctively stimulated during the highly pathogenic H5N8 virus infection in mice, suggesting a potential universal role of NONMMUT011061 in the pathogenesis of different H5 IAV. Altogether, these results provide a subset of lncRNAs that might play important roles in the pathogenesis of influenza virus and add the lncRNAs to the vast repertoire of host factors utilized by IAV for infection and persistence.

## Introduction

The primary natural host reservoir of highly pathogenic avian influenza (HPAI) H5N1 virus is wild waterfowl. However, these viruses can occasionally infect other host species, including wild birds, terrestrial poultry, various mammals and even human beings. As of March 2 2018, 860 laboratory-confirmed human cases of H5N1 infection, including 454 deaths, have been reported (http://www.who.int). Although pathogenesis of the H5N1 subtype has been extensively studied, molecular events leading to disease are still obscure. Using transcriptomics or proteomics profiling, accumulated studies have demonstrated that an aberrant immune response contributes to the development of typical pneumonia and subsequent acute respiratory distress syndrome (ARDS), which ultimately lead to death [1–4]. However, the underlying host molecular mechanisms causing the aberrant host response are largely unknown.

Traditional studies examining the host transcriptional response to pathogen infection mainly focus on protein-coding genes. However, the majority of the mammalian genome is transcribed into non-coding RNAs (ncRNAs), including the small ncRNAs (< 200 bp) and long ncRNAs (lncRNAs) (> 200 bp)[5]. LncRNAs are the major regulators of gene expression and are involved in multiple biological processes, including genomic imprinting, embryonic development, cell differentiation, tumor metastasis and regulation of cell cycle [6–10]. LncRNAs regulate gene expression at the epigenetic, transcriptional and post-transcriptional levels in diverse biological contexts[6]. In addition, a variety of molecular mechanisms were used as major regulators of these function, such as modulating histone modifications[11], interfering the transcription [12,13], regulating patterns of alternative splicing [14,15],generating small RNAs[16], and modulating protein activation and localization[17].

Although accumulated studies have identified the functional importance of lncRNAs in innate immune response [13,18–24] and host-pathogen interactions [25–29], the specific function of lncRNAs in innate immune response to influenza A virus (IAV) infection remains largely unexplored. As far as we known, only a few lncRNAs, such as NRAV (negative regulator of antiviral response)[11], NEAT1 (nuclear enriched abundant transcript 1)[30], Bst2/BISPR (bone marrow stromal cell antigen 2 IFN-stimulated positive regulator) [31,32] and VIN (virus inducible lincRNA) [33] have been demonstrated to be associated with the innate immunity against influenza virus and viral pathogenesis. Therefore, studies are still needed to better understand the potential roles of other lncRNAs in the pathogenesis of influenza virus.

Our previous study has characterized two HPAI H5N1 viruses, CK10 and GS10, that have similar genetic background but differ greatly in pathogenicity in mice[34]. CK10 is a highly pathogenic strain, as evidenced by the extremely low lethal dose in mice (MLD_50_: 0.33 log_10_ 50% embryo infectious dose, EID_50_), whereas GS10 is low pathogenic (MLD_50_ > 6.32 log_10_ EID_50_). Transcriptomics analysis showed that CK10 elicited a more potent innate immune response in the mouse lung compared to GS10[35]. Further quantitative proteomics suggested that an early intense host response associated with the lung injury to CK10 may contribute to the high virulence of this virus in mice[36]. In this study, to evaluate the role of lncRNAs in the pathogenesis of influenza virus of the H5N1 subtype, we systematically compared the expression profile of lncRNAs in the lung of mice infected with CK10 or GS10 using the RNA deep-sequencing technology. Our results demonstrated that these two viruses distinctively regulated the expression of numerous lncRNAs, suggesting that these lncRNAs may be a new class of regulatory molecules involving in determining the outcome of H5N1 virus infection.

## Materials and methods

### Ethics statement

This study was carried out in strict accordance with the recommendations in the Guide for the Care and Use of Laboratory Animals of the Ministry of Science and Technology of the People’s Republic of China. The protocols for animal experiments were approved by the Jiangsu Administrative Committee for Laboratory Animals (approval number: SYXK-SU-2007–0005), and complied with the guidelines of Jiangsu laboratory animal welfare and ethics of Jiangsu Administrative Committee of Laboratory Animals. All experiments involving live viruses and animals were housed in negative-pressure isolators with HEPA filters in bio-safety level 3 (BSL3) animal facilities in accordance with the institutional bio-safety manual.

### Viruses

A/Chicken/Jiangsu/k0402/2010 (H5N1) (CK10) was isolated from a dead chicken in 2010, and A/Goose/Jiangsu/k0403/2010 (H5N1) (GS10) was isolated from an apparently healthy goose in live poultry market[37]. CK10 is highly pathogenic in mice (MLD_50_ = 0.33 log_10_ EID_50_), whereas GS10 is low pathogenic (MLD_50_ > 6.32 log_10_ EID_50_) in this animal model[34]. The entire genome of the two viruses differed by 30 amino acid (aa) distributed throughout eight genes. [34] Highly pathogenic strain of H5N8 subtype, A/goose/Jiangsu/QD5/2014 (QD5), was isolated from swab samples from apparently healthy geese at a wholesale live-bird market in 2014[38]. QD5 is also highly pathogenic in mice, with a MLD_50_ of 2.83 log_10_ EID_50_[38]. Viruses were plaque-purified three times in MDCK cells and propagated once in specific-pathogen-free (SPF) embryonated chicken eggs.

### Mouse experiment

To collect lung samples for deep-sequencing, groups of fourteen 6-week-old female BALB/c mice were infected intranasally (i.n.) with 10^5.0^. EID_50_ of CK10 or GS10, respectively. Another group of mice were i.n. inoculated with 50 μl of PBS as mock control. At day 1, 3 and 5 post inoculations (p.i.), three mice from each group were euthanized and the lungs were collected for lncRNAs quantification, cytokine profiling, histopathological examination and virus load measurement. The remaining five mice of each group were monitored daily for weight loss and survival for 14 days.

To identify the potential role of the selected lncRNA in IAV pathogenesis, groups of nine 6-week-old female BALB/c mice were infected i.n. with 10[5].° EID_50_ of CK10 (H5N1), QD5 (H5N8) or GS10 (H5N1), respectively. At day 1, 2 and 3 p.i., three mice from each group were euthanized and the lungs were collected for determination of lncRNAs expression and viral replication.

### Poly (A)-independent and strand-specific RNA-sequencing

The lung samples were homogenized in TRIzol (Invitrogen, CA, US) using the MagNA Lyser system (Roche) according to the manufacturer’s instructions. RNA was further purified using the miRNeasy minikit (Qiagen) based on the manufacturer’s instructions. The purity of the RNA samples was verified spectroscopically, and RNA quality was assessed using Agilent Bioanalyzer 2100[39]. Only samples with an RNA integrity number greater than 8 were used. rRNA was depleted from 1 mg of total RNA using RiboZero (Illumina). cDNA libraries were prepared from the remaining RNA, without poly (A) selection, using the TruSeq Stranded RNA LT kit (Illumina) following the TruSeq stranded total RNA sample preparation guide provided by the vendor (RS-122-9007DOC). The libraries underwent cluster generation using TruSeq PE Cluster Kit v3-cBot-HS and 100 cycles of paired-end sequencing using TruSeq SBS Kit v3-HS (Illumina) and an Illumina HiSeq 2000 sequencer as described previously [39,40].

### Analysis of RNA-sequencing data

After sequencing, the raw reads that were generated by sequencers, were saved in the fastq format. To obtain reliable clean reads, the dirty raw reads were filtered according to six criteria based on fastx software(version:0.0.13): (i) reads with sequence adaptors were removed; (ii) reads with more than 5% ‘N’ bases were removed; (iii) reads with length < 20 bases were removed; (iv) 3ʹ end of Q (Q = −10 log error ratio) less than 10 of the base quality were removed; (v) low-quality reads, in which less than 50% of the quality were > 20 bases were removed; (vi) and ribosomal RNA sequences that were obtained from the ribosomal RNA database SILVA (http://www.arb-silva.de/) by the software SOAP v2.2.0 [41] were removed based on an allowance of no more than three mismatched bases. All subsequent analyses were based on clean reads. Tophat v2.0.9 (http://tophat.cbcb.umd.edu/) spliced mapping was used to map the cleaned reads to the mouse mm10 reference genome with two mismatches.

The clean reads that were uniquely mapped to lncRNAs were used to calculate the expression levels. After genome mapping, Cufflinks v2.1.1 (http://cufflinks.cbcb.umd.edu/) was run with a reference annotation to generate Fragments Per Kilobase of exon model per Million mapped reads (FPKM) values for known gene models. Differentially expressed genes were identified using Cuffdiff, implemented in Cufflinks[42]. The relative expression levels of lncRNAs in the CK10 or GS10 and mock control groups were measured as the number of uniquely mapped FPKM. The formula was defined as follows: FPKM = 10^9^× C/(NL × 10^−3^), where C was the number of reads that uniquely mapped to the given transcript, N was the number of reads that uniquely mapped to all transcripts, and L was the total length of the given transcript. The FPKM method eliminates the influences of different transcript lengths and sequencing discrepancies on the calculation of expression. Therefore, the FPKM value was directly used to compare the differences in lncRNA expression between the samples. The fold change from the normalized expression was calculated as FPKM CK10 or GS10/FPKM mock to assess the levels. Genes satisfying the condition of FPKM CK10 or GS10 value/FPKM mock value > 1.5 were defined as those up-regulated in virus-infected group, whereas genes satisfying the condition of FPKM CK10 or GS10 value/FPKM mock value < 0.67 were defined as those down-regulated in virus-infected group. To compensate for false-positive findings at each significance threshold, the *p*-value significance threshold in multiple tests was further set by the false discovery rate (FDR)[43]. Therefore, we identified lncRNAs that were differentially regulated between the CK10, GS10 and mock control groups based on the following criteria: *p* < 0.05, FDR < 0.05 and absolute value of the fold change > 1.5.

### Identification and expression analysis of lncRNAs

Cufflinks was used to assemble reads into transcripts. Novel transcripts were obtained after comparing all the assembled transcript isoforms with the mouse known protein coding transcripts using Cuffcompare[42]. Putative lncRNAs were defined as novel transcripts set through the following filters: length ≥ 200 bp; number of exons ≥ 2; ORF ≤ 300 bp; no or weak protein coding ability (CPC score < 0, CNCI score < 0 and no significant similarity with Pfam database)[44]. Finally, to generate a unique set of lncRNAs, we used Cuffcompare to integrate the RNA-seq derived lincRNAs with the known lncRNAs previously annotated by NONCODE V4.0 (http://www.noncode.org/). Differentially expressed lncRNAs were selected for target prediction. The genes transcribed within a 10 kbp window upstream or downstream of lncRNAs were considered as *cis*-acting target genes. The *trans*-acting target genes were predicted using RNAplex software[45].

### Real-time PCR analysis of lncRNAs and mRNAs expression

Quantitative real-time PCR (qRT-PCR) was used to validate the expression of lncRNAs and mRNAs identified by RNA-sequencing analysis. Briefly, total RNA was isolated from the tissues using the TRIzol reagent and treated with DNase I (Invitrogen). A total of 1 μg of RNA per sample was reverse transcribed into cDNA using 400 U RevertAid Premium Reverse Transcriptase and 100 μM random primers in the presence of RNase inhibitor at 50°C for 30 min. The reaction mixture contained cDNA, 200 nM of each primer and 10 μl of 2 × SYBR Green PCR Master Mix (Takara, Shiga, Japan). PCR reactions were performed in triplicate using the ABI Prism 7300 system (Applied Biosystems, CA, US) with the following cycle profile: 1 cycle at 50°C for 2 min and 1 cycle at 95°C for 5 s followed by 40 cycles at 95°C for 5 s and 60°C for 31 s. 1 cycle for melting curve for all reactions was added to verify product specificity. Expression value of each gene, relative to the GAPDH, was calculated using the equation 2^−ΔΔ*C*t^ method.

### Construction of the LncRNA/mRNA Coexpression Network

To construct the lncRNA/mRNA coexpression network, we calculated the Pearson correlation coefficient and R value to evaluate lncRNA-mRNA correlation[46]. The network construction procedure includes: (1) Preprocess data: the same mRNAs with different transcripts taking the median value represent the gene expression values, without special treatment of lncRNAs expression value. (2) Screen data: remove the subset of data according to the lists showing the differential expression of lncRNAs and mRNAs. (3) Calculate the Pearson correlation coefficient and use R value to calculate the correlation coefficient between lncRNAs and mRNAs. (4) Screen by Pearson correlation coefficient: select the Pearson correlation coefficient ≥ 0.99 or ≤ – 0.99 as the meaningful value and draw the lncRNA/mRNA coexpression network by using the cytoscape program.

### Accession numbers

All primary RNA-sequencing data have been deposited in the Gene Expression Omnibus (GEO) database (http://www.ncbi.nlm.nih.gov/geo/info/linking.html) under accession number GSE100522. The genomic sequences of the CK10 and GS10 viruses are available in GenBank under accession numbers JQ638673 to JQ638688.

### Statistical analysis

Viral loads are expressed as the mean ± standard deviation (SD) from three individuals. Cytokine levels and lncRNA expression levels are expressed as the mean fold change ± standard error (SE) of the mean from three individuals. Statistical analyzes were performed using Independent-Sample T test.

## Results

### Pathogenicity of CK10 and GS10 in mice

**Figure 1.**
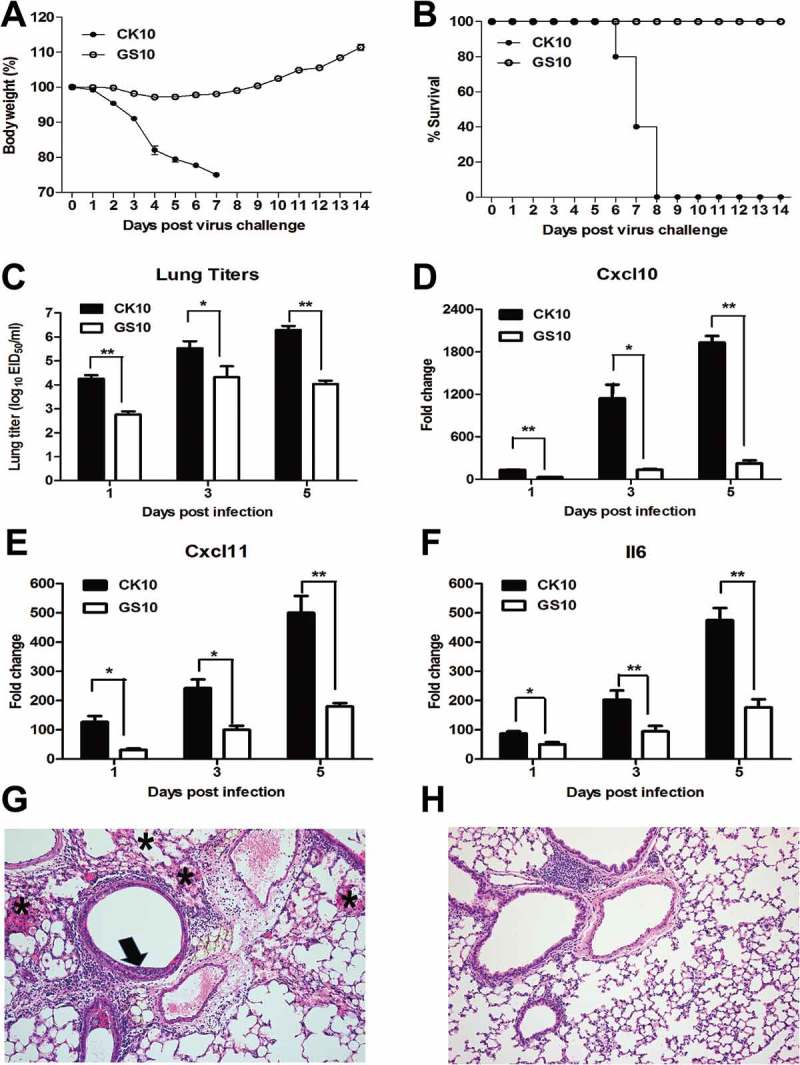
Pathogenicity of CK10 and GS10 in mice. (**A**) Mean weight loss of mice infected with 10^5.0^[5]. EID_50_ of CK10 and GS10 viruses (*n *= 5). Mice were humanely killed when they lost ≥ 25% of their initial body weight. *Error bar* represents stand deviation (SD). (B) Survival rate of mice infected with the indicated viruses (*n *= 5). (**C)** Viral replication in the mouse lung. Values shown are the mean ± SD of the results from five individuals (*p < 0.05). *Asterisk* indicates significant difference between the CK10 and the GS10 virus. (D) – (F) Cytokines and chemokines expression in the mouse lung. Levels of cytokine or chemokine were expressed as the mean fold change ± standard error (SE) of the mean. *p < 0.05 and **p < 0.01, *asterisk* or *double asterisk* indicates significant difference between CK10 and GS10. (G) and (H) Representative histopathological changes in H&E (hematoxylin and eosin)-stained lung tissues on day 1 p.i.. (G) CK10 virus-infected mouse lung. Pulmonary alveolar hemorrhage (shown as *asterisk*), bronchial mucosa injury and accompany by inﬂammatory cells infiltration around the bronchus (shown as *black arrow*). (H) GS10 virus-infected mouse lung. No obvious histopathology was observed in the GS10 virus-infected mouse lung.

To systematically compare the pathogenicity of this pair of viruses, groups of fourteen mice were infected i.n. with 10^5.0^. EID_50_ of CK10 or GS10. At day 1, 3 and 5 p.i., three mice from each group were euthanized and the lungs were collected for determination of transcriptional cytokine response and viral replication. The remaining five mice of each group were monitored daily for weight loss and survival rate over the course of infection. As shown in Figure 1(A), mice infected with CK10 virus showed more severe weight loss compared with that of GS10 virus. In addition, no death was found in GS10-infected mice within a 14-day observation period, while all the CK10-infected mice succumbing to death by day 8 p.i. (Figure 1(B)). Moreover, CK10 replicated at significantly higher titers in mouse lung than GS10 at all-time points (Figure 1(C)). According to our previous study [35] and the established knowledge about the role of *Cxcl10, Cxcl11* and *Il6* in inflammation, we determined the expression of these cytokines and found that CK10 stimulated significantly higher expression levels of these genes compared to GS10 in the mouse lung (Figure 1D-F). Mean while, we also compared the mouse lung histopathology induced by these pair of viruses. As shown in Figure 1, we can see that early at day 1 p.i., the highly pathogenic CK10 virus induced a more severe lung injury than the non-virulent GS10 virus, represented by pulmonary alveolar hemorrhage, bronchial mucosa injury and accompanied by inﬂammatory cells infiltration around the bronchus (Figure 1(G)). However, no obvious histopathology was observed in the GS10 virus-infected mouse lung (Figure 1(H)). Altogether, these results demonstrated that CK10 virus is more virulent than GS10 virus in mice and is associated with higher lung titer and proinflammatory cytokine expressions.

### Whole-transcriptome analysis of mouse lung infected with CK10 and GS10

**Figure 2.**
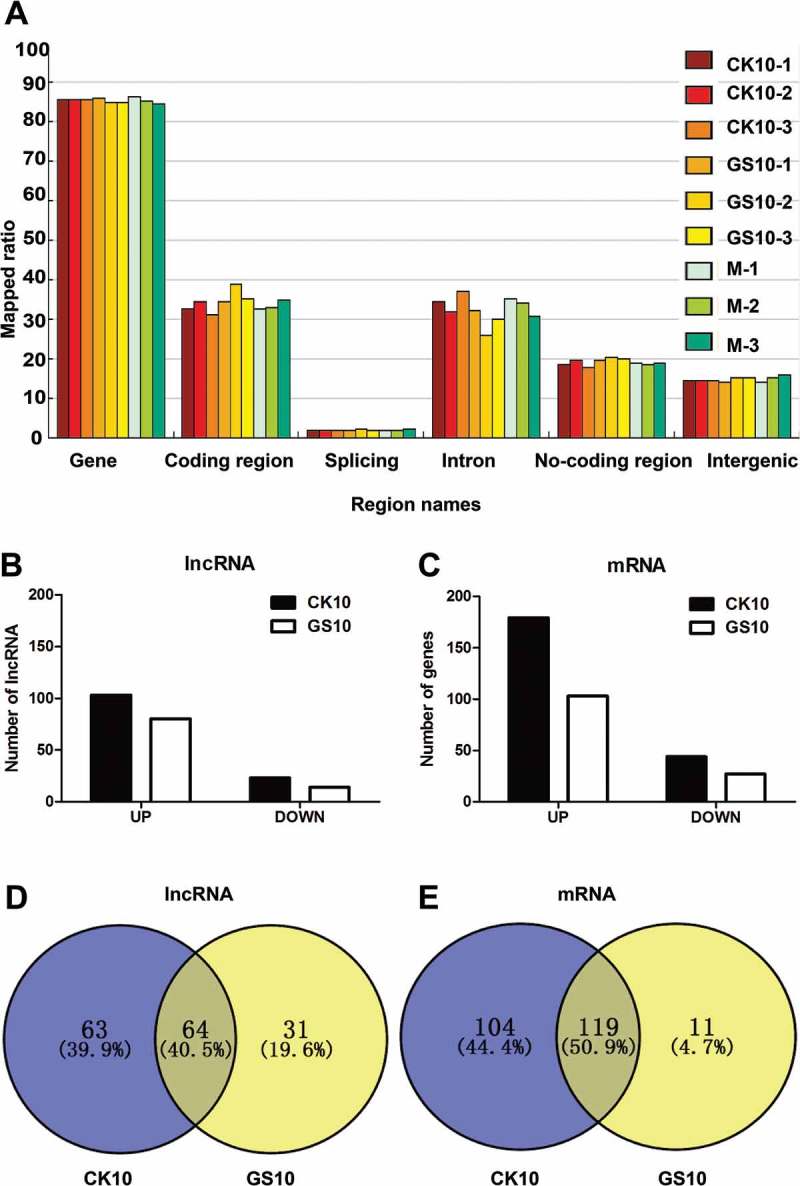
Analysis of the lncRNA data. (A) Region distribution of the tested reads. Results shown are the region distribution of the tested reads, including gene, coding region, splicing, intron, non-coding region and intergenic. Among them, 5-UTR, 3-UTR, non-coding RNA regions are covered in non-coding region. (B) Numbers of significantly differentially expressed (SDE) lncRNAs in the process of infection with CK10 or GS10 relative to mock (*p *< 0.05, fold change > 1.5 or < 0.67). (C) Numbers of SDE mRNAs in the process of infection with CK10 or GS10 relative to mock (*p *< 0.05, fold change > 1.5 or < 0.67). (D) Venn diagram showing the distribution of SDE lncRNAs in the process of infection with CK10 or GS10. (E) Venn diagram showing the distribution of SDE mRNAs in the process of infection with CK10 or GS10 viruses.

Since the pathogenicity study showed that the two viruses exhibited a remarkable difference in replication, cytokine response and histological changes early at day 1 p.i. (Figure 1), we then performed a whole-transcriptome analysis of the collected lung samples at this time point using next-generation sequencing (NGS). Mapped ratio of region distribution of all the tested reads, including gene, coding region, splicing, intron, non-coding region, were shown in Figure 2(A). Among them, a total of 1,990 novel lncRNAs covering three major types of lncRNAs (Intergenic, Intron and Antisense) were identified. In addition, CK10 virus induced expression of 48,759 known lncRNAs and 19,491 mRNAs, whereas the corresponding numbers for GS10 virus were 49,411 and 19,843, respectively. Among these RNAs, 126 lncRNAs regulated by CK10 displayed significantly altered expression levels compared with the mock control, including 103 up-regulated lncRNAs (fold change: CK10 vs. mock > 1.5, *p* < 0.05, FDR< 0.05) and 23 down-regulated lncRNAs (fold change CK10 vs. mock > −1.5, *p* < 0.05, FDR< 0.05) (Figure 2(B)). As for the GS10 group, a total of 94 lncRNAs were significantly differentially expressed (SDE), with 80 up-regulated and 14 down-regulated (Figure 2(B)). Moreover, CK10 induced 223 SDE mRNAs (fold change CK10 vs. mock > 1.5 or −1.5, *p* < 0.05, FDR< 0.05), while only 130 SDE mRNAs were screened for GS10 (Figure 2(C)). Interestingly, among these SDE RNAs, 64 lncRNAs (40.5%) and 119 mRNAs (50.9%) were shared by the two viruses (Figure 2(D – E)). Therefore, these results showed that early at day 1 p.i., CK10 induced a larger number of SDE RNAs than GS10, and there was a partial of SDE lncRNAs and mRNAs overlapped between these two groups.

### lncRNA/mRNA coexpression network

**Figure 3.**
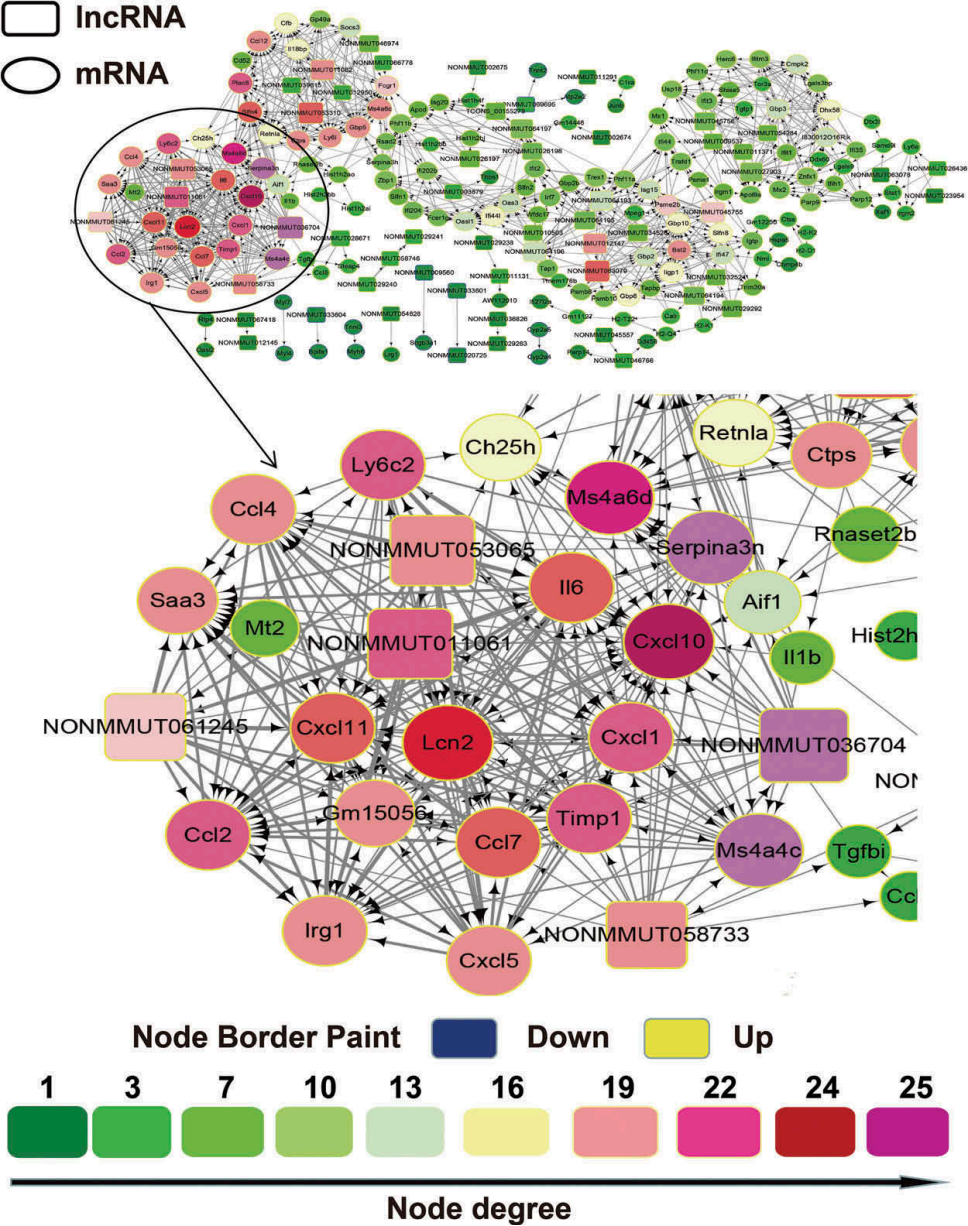
The lncRNA/mRNA coexpression network constructed using the cytoscape program for the CK10 group. The lncRNAs and mRNAs with Pearson correlation coefficients ≥ 0.99 or ≤ −0.99 were selected to draw the regulatory network using the cytoscape program. In gene-coexpression networks, each gene corresponds to a node. Two genes are connected by an edge, indicating a strong correlation. Within the network analysis, a degree is the simplest, most important measure of the centrality of a gene within a network and determines the relative importance. A degree is defined as the number of directly linked neighbors. In the network, the node size indicates the node degrees and the number represents the number of directly linked neighbours that are associated with each color. Therefore, the larger the node size suggested the targeted lncRNA or mRNA could directly linked with more neighboring genes.

Although accumulating studies have attempted to reveal the functional significance of lncRNAs, the biological roles of most lncRNAs are still unknown. Biological processes and cellular regulation networks are very complex, involving the interactions of various molecules, such as proteins, RNAs, and DNAs. Our RNA-sequencing data not only provided the information of lncRNAs expression, but also provided mRNAs expression patterns in the virus or mock-inoculated mouse lung. We thus constructed an lncRNA/mRNA coexpression network based on the SDE RNAs between CK10 or GS10 virus and mock control and investigated the potential interaction between mRNAs and lncRNAs of each virus-infected group. The coexpression network for the CK10 group was composed of 56 differentially expressed lncRNAs, 149 differentially expressed mRNAs and 205 network nodes (Figure 3). The network showed that several lncRNAs (NONMMUT036704, NONMMUT011061, NONMMUT053065, NONMMUT058733 and NONMMUT061245) correlated with a great number of targeted mRNAs, and vice versa (Figure 3). This coexpression network also indicated that one lncRNA (NONMMUT036704) could target 22 network nodes and one coding gene (C*xcl10*) could target 25 network nodes. In addition, the second ranked lncRNA (NONMMUT011061) and mRNA (Cxcl11), could both target 21 network node. As for the GS10 group, the coexpression network was composed of 65 lncRNAs, 102 mRNAs and 167 network nodes (**Figure S1**). However, as shown in **Figure S1**, in the GS10-infected mouse lung, the lncRNAs were not highly correlated with the mRNAs and vice versa. Taken together, these results suggested the closer inter-regulation of lncRNAs and mRNAs in the early stage of CK10 virus infection compared with GS10 virus.

### Biofunction analysis of the lncRNAs-coexpressed mRNAs

**Figure 4.**
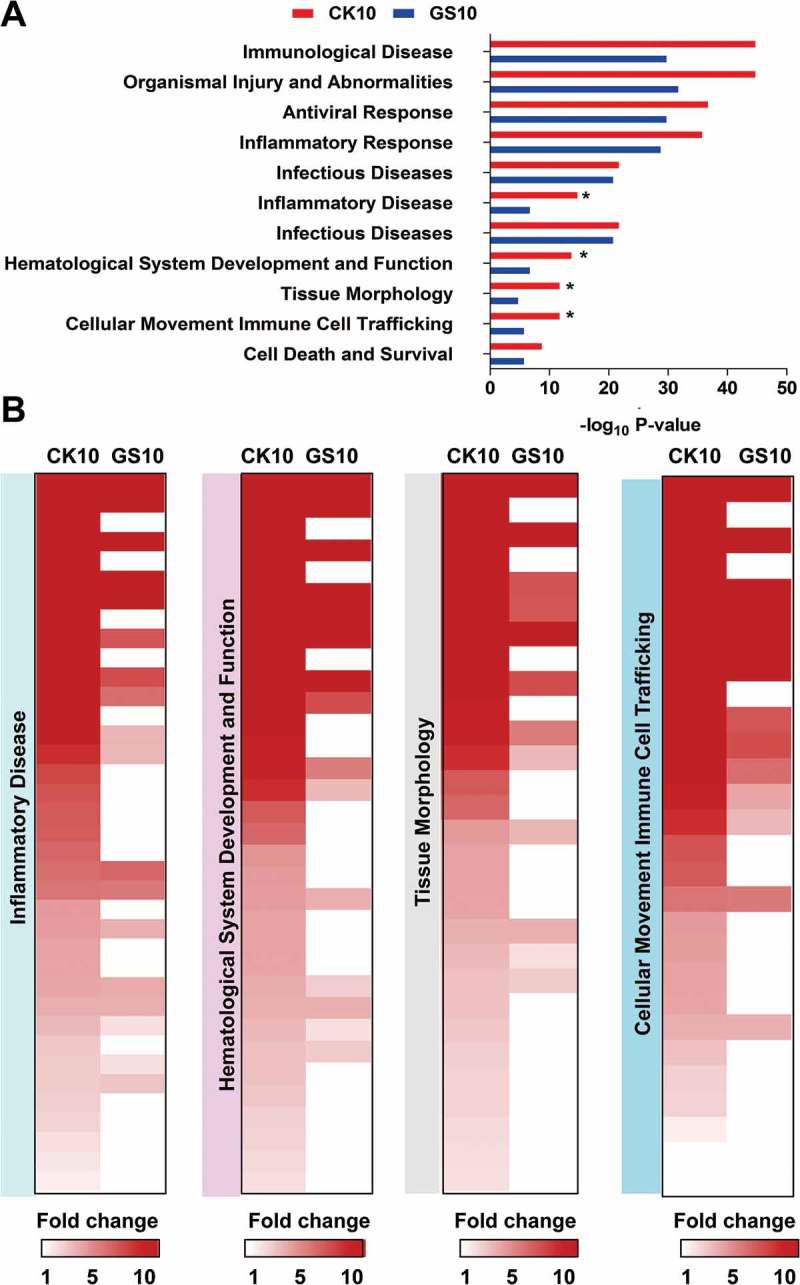
Disease and bio-functional categories of the lncRNAs-coexpressed mRNAs induced by CK10 or GS10 by IPA analysis. (A) Important bio-functions associated with the lncRNAs-coexpressed mRNAs. ‘*’, indicates the P value of the CK10 virus-infected group was above 2-fold than that of GS10. (B) Crucial bio-functions related to inﬂammatory responses that were highly induced by CK10.

To predict the roles of the selected differentially expressed lncRNAs in response to CK10 and GS10, the lncRNAs-coexpressed mRNAs were then imported into the Ingenuity Pathways Analysis (IPA) software (Ingenuity Systems, Redwood, CA, US) for bio-function category construction. As a result, these mRNAs mainly clustered into some important functional groups, such as ‘Immunological Disease’, ‘Organismal Injury and Abnormalities’, ‘Antiviral Responses’ and ‘Inflammatory Response’ (Figure 4(A)). Among of them, ‘Antiviral Responses’ and ‘Inflammatory Response’ were intensely activated (activation z-score above 2.0) both by the CK10 virus and GS10, however, more genes involved in these functions in the CK10 virus-infected group. Particularly, the lncRNAs-coexpressed mRNAs associated with ‘Inflammatory Disease’, ‘Hematological System Development and Function’, ‘Tissue Morphology’ and ‘Cellular Movement and Immune Cell Trafficking’ were more than 2-fold induced (according to the -log_10_ P-value) in the lungs in CK10-infected mice than that of GS10 (which indicated as ‘*’) (Figure 4(A)). Moreover, a number of genes were more upregulated in these functions by CK10 virus than that of GS10 virus (Figure 4(B)). Therefore, these results suggested that the SDE lncRNAs-coexpressed mRNAs activated by CK10 were highly related with the aberrant and uncontrolled inflammatory related responses.

### Canonical pathway analysis of the lncRNAs-coexpressed mRNAs

**Figure 5.**
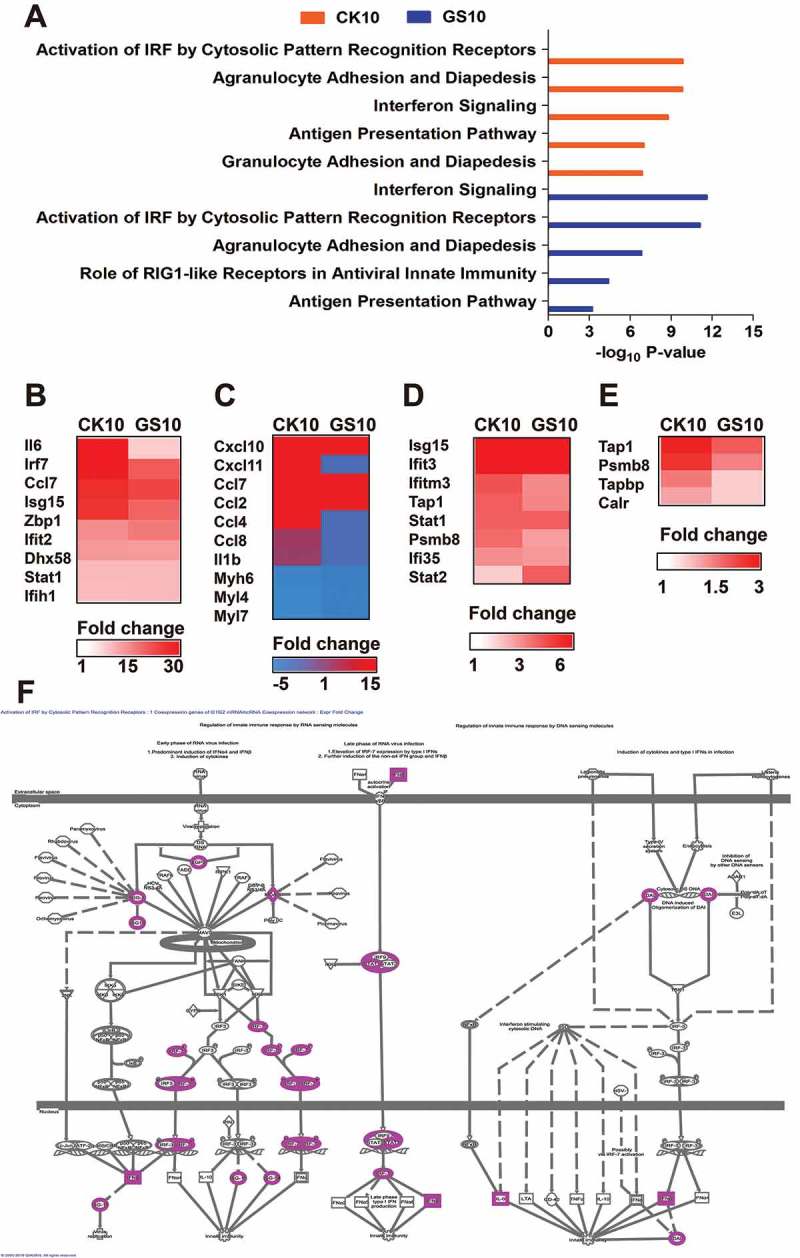
Canonical pathways of the lncRNAs-coexpressed mRNAs stimulated by CK10 or GS10 by IPA analysis. (A) The top canonical pathways associated with the lncRNAs-coexpressed mRNAs. (B) The expression profiles of the lncRNAs-coexpressed mRNAs related to the ‘Activation of IRF by Cytosolic Pattern Recognition Receptors’ top 1 pathway for the CK10 group. (C) The expression profiles of the lncRNAs-coexpressed mRNAs related to the ‘Agranulocyte Adhesion and Diapedesis’ top 2 pathway for the CK10 group. (D) The expression profiles of the lncRNAs-coexpressed mRNAs related to the ‘Interferon Signaling’ top 3 pathway for the CK10 group. (E) The expression profiles of the lncRNAs-coexpressed mRNAs related to the ‘Antigen Presentation Pathway’ top 4 pathway for the CK10 group. (F) The detail presentation of top 1 canonical pathway ‘Activation of IRF by Cytosolic Pattern Recognition Receptors’ induced by CK10.

To further gain insight into the function of the lncRNAs-coexpressed mRNAs, we next compared the top 5 canonical pathways (generated by IPA) associated with these SDE mRNAs. The results showed that the top 5 canonical pathways (according to the -log_10_ P-value) for CK10 virus were ‘Activation of IRF by Cytosolic Pattern Recognition Receptors’, ‘Agranulocyte Adhesion and Diapedesis’, ‘Interferon Signaling’, ‘Antigen Presentation Pathway’ and ‘Granulocyte Adhesion and Diapedesis’ was hyper-induced in CK10-infected mice (Figure 5(A)). Among of them, ‘Activation of IRF by Cytosolic Pattern Recognition Receptors’ and ‘Interferon Signaling’ pathways were highly activated (activation z-score above 2.0). In contrast, the top 5 canonical pathways of GS10 were ‘Interferon Signaling’, ‘Activation of IRF by Cytosolic Pattern Recognition Receptors’, ‘Agranulocyte Adhesion and Diapedesisy’, ‘Role of RIG1-like Receptors in Antiviral Innate Immunity’ and ‘Antigen Presentation Pathway’, respectively (Figure 5(A)). Therefore, there were highly overlapped between the SDE lncRNAs-coexpressed mRNAs-mediated top canonical pathway in CK10 and GS10 virus-infected mouse lung. However, further analysis demonstrated that mice infected with the CK10 virus exhibited an overall stronger expression of lncRNAs-coexpressed mRNA associated with these pathways, including ‘Activation of IRF by Cytosolic Pattern Recognition Receptors’ (Figure 5(B – F)),

‘Agranulocyte Adhesion and Diapedesis’ (Figure 5(C)), ‘Interferon Signaling’ (Figure 5(D)) and ‘Antigen Presentation Pathway’ (Figure 5(E)). Therefore, these results indicate the SDE lncRNAs-coexpressed mRNAs induced by CK10 and GS10 shared some similarities in association with the inflammatory response related pathways; however, CK10-regulated mRNAs were more upregulated in these pathways.

### qRT-PCR validation of the selected lncRNAs and lncrnas-coexpressed mRNA

**Figure 6.**
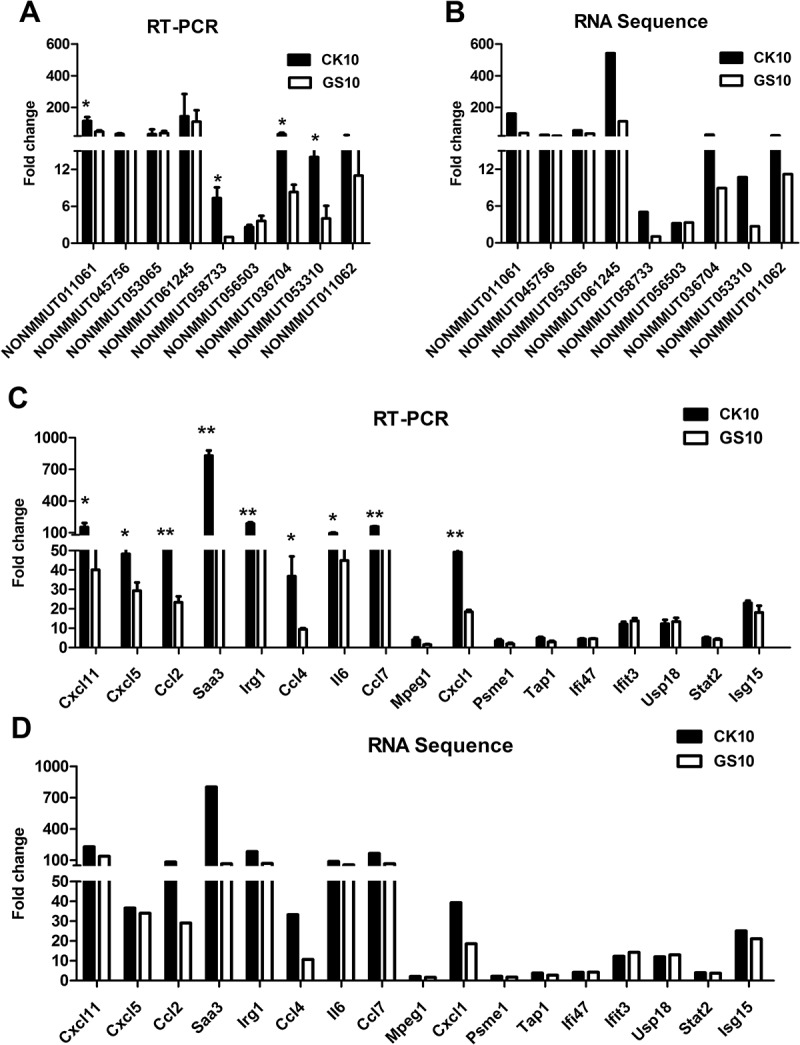
Validation of the RNA-sequencing data. (A) Alteration of the expression of the selected lncRNAs in CK10- or GS10-infected mouse lungs at 24 h p.i. was analyzed by qRT-PCR. Values shown are the mean fold change ± SE of the results from three individuals (*p < 0.05). *Asterisk* indicates a significant difference between CK10 and GS10. (B) RNA-sequencing results of the targeted lncRNAs were shown as control. (C) Alteration of the expression of the selected mRNAs in CK10- or GS10-infected mouse lungs at 24 h p.i. was analyzed by qRT-PCR. Values shown are the mean ± SD of the results from three individuals (*p < 0.05, **p < 0.01). *Asterisk* indicates a significant difference between CK10 and GS10. (D) RNA-sequencing results of the selected mRNAs were shown for comparison.

To verify the RNA-sequencing data, a subset of RNAs in replicate samples was examined using qRT-PCR. Two categories of RNA were selected: 9 lncRNAs that were highly coexpressed with the annotated protein-coding genes and 17 mRNAs that were tightly related with the targeted lncRNAs. Pearson correlation analysis was applied to measure the significance of the correlations. As a result, a good correlation between RNA deep-sequencing data and qRT-PCR results on the set of independent samples with multiple replicates was observed (**Figure 6 and Figure S2**). The analysis results revealed a highly statistically significant (p < 0.001) correlation between the qRT-PCR and RNA-sequence data and the correlation coefficient were all above 0.8 (**Figure S2**). QRT-PCR data also showed that the expression of NONMMUT036704, NONMMUT011061, NONMMUT058733 and NONMMUT053310 in CK10-infected mice was significantly higher than that in GS10-infected animals (**Figure 6(A),** Supplementary information 1 listed the sequence information for NONMMUT011061). In addition, some mRNAs that highly correlated with NONMMUT011061, including *Cxcl11, Cxcl5, Ccl2, Saa3, Irg1, Ccl4, Il6, Ccl7* and *Cxcl1*, were also activated to significantly higher levels compared to those of the GS10 group (Figures 3 and 6(C)).

### Potential relevance of NONMMUT011061 with virulence of H5 IAV in mice

**Figure 7.**
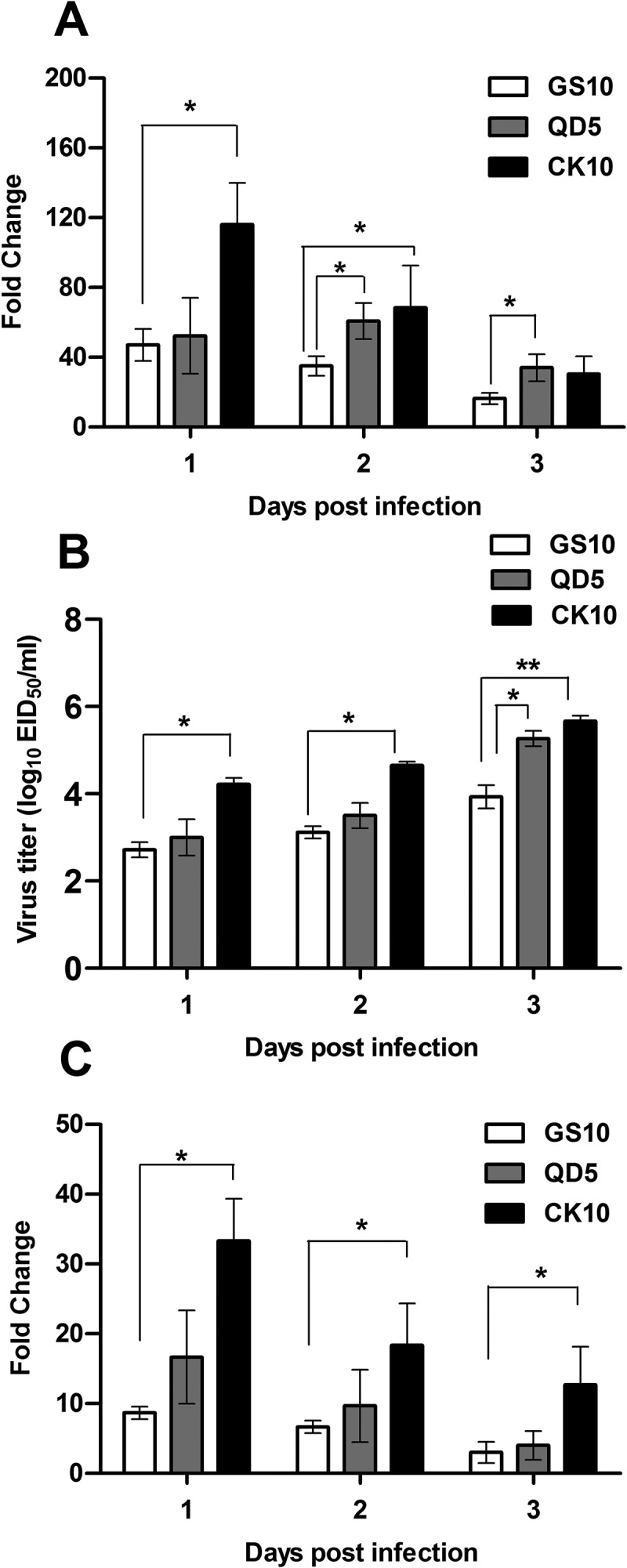
Potential relevance of NONMMUT011061 with virulence of H5 IAV in mice. Groups of nine mice were infected with CK10, GS10 or QD5 (a H5N8 strain that is also highly pathogenic in mice). (A) The expression pattern of NONMMUT011061 was determined in the mouse lung at day 1, 2 and 3 p.i. Values are expressed as the mean fold change ± SE of the mean from three individuals. (B) Virus titers in the lungs. Values shown are the mean ± SD of the results from three individuals (*p < 0.05, **p < 0.01). *Asterisk* indicates a significant difference between highly pathogenic viruses (CK10 or QD5) and GS10. (C) The expression pattern of NONMMUT036704 was determined in the mouse lung at day 1, 2 and 3 p.i. Values are expressed as the mean fold change ± SE of the mean from three individuals.

Our coexpression network indicated that one NONMMUT036704 could target 22 network nodes and lncRNA NONMMUT011061 could target 21 network nodes (Figure 3). Moreover, a number of inflammatory genes which were highly correlated with NONMMUT011061 were highly activated during the highly pathogenic virus infection (Figures 3 and 6(C)). Therefore, to reveal the potential role of NONMMUT011061 and NONMMUT036704 in the pathogenesis of H5 IAV, groups of mice were inoculated with different H5 viruses (H5N1: CK10 and GS10; H5N8: QD5) [38] and the expression pattern of NONMMUT011061 and NONMMUT036704 in the mouse lung were determined at day 1, 2 and 3 p.i. The results showed that the expression of NONMMUT011061 and NONMMUT036704 in CK10-infected mice was significantly higher than that of GS10 at several time points (Figure 7(A and C)). Meanwhile, CK10 replicated to significantly higher levels than GS10 at all three time points (Figure 7(B)). More importantly, the highly pathogenic H5N8 virus QD5 also stimulated significantly higher expression level of NONMMUT011061 than that of GS10 at day 2 and 3 p.i. However, although significantly higher virus load was also observed in QD5-infected mice compared to that in GS10-infected mice at day 3 p.i., NONMMUT036704 is not significantly stimulated by the highly pathogenic H5N8 virus in mice (Figure 7(C)). Taken together, these results showed that the NONMMUT011061 was distinctively stimulated during the highly pathogenic H5N1 and H5N8 virus infection in mice, suggesting a potential role of NONMMUT011061 in the pathogenesis of different H5 IAV.

### Bioinformatics analysis of NONMMUT011061

**Figure 8.**
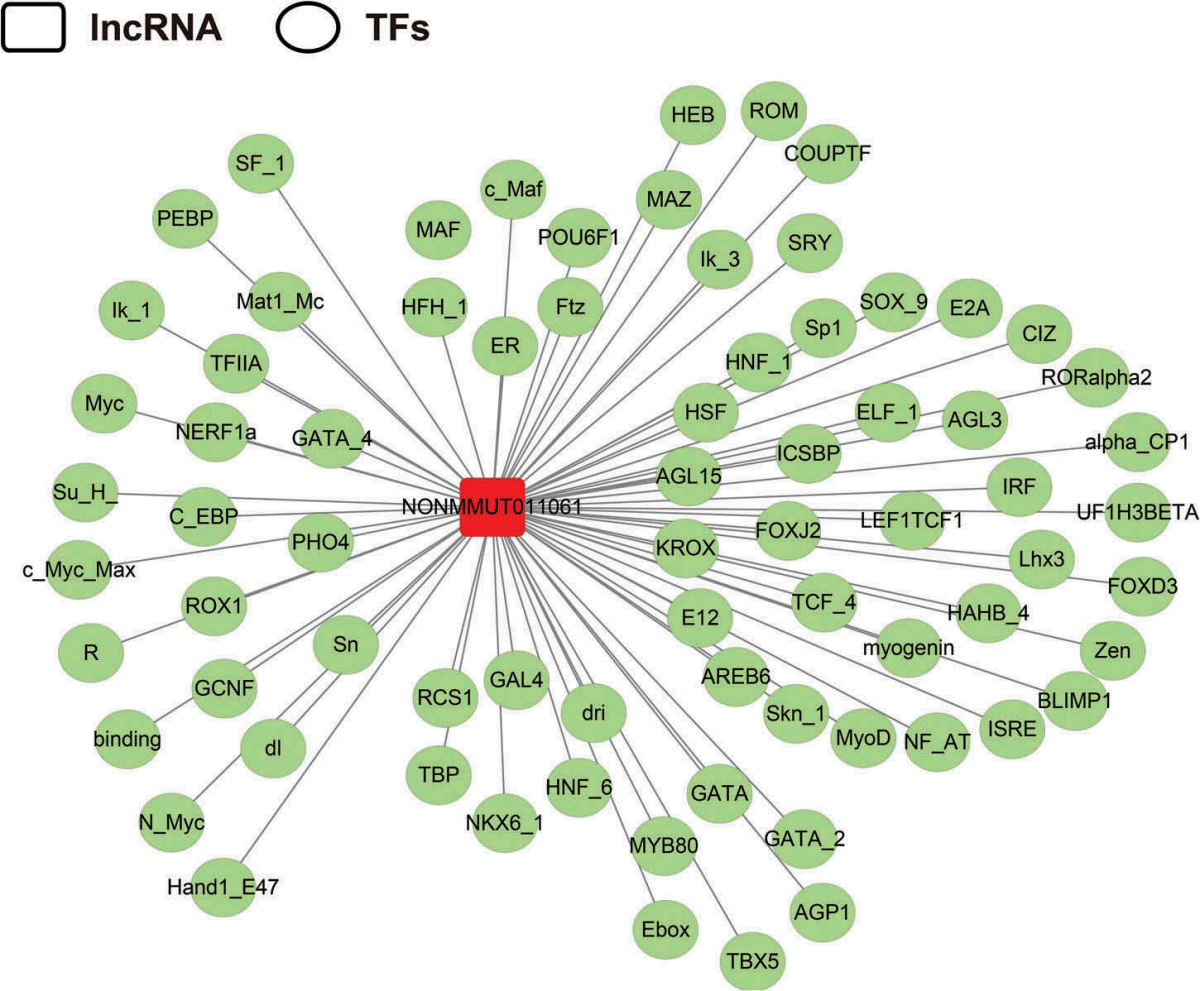
Prediction of the potential transcriptional factors (TFs) of the NONMMUT011061. The TRANSFAC database was used to predict the TFs associated with NONMMUT011061 (http://www.gene-regulation.com/index2.html).

**Figure 9.**
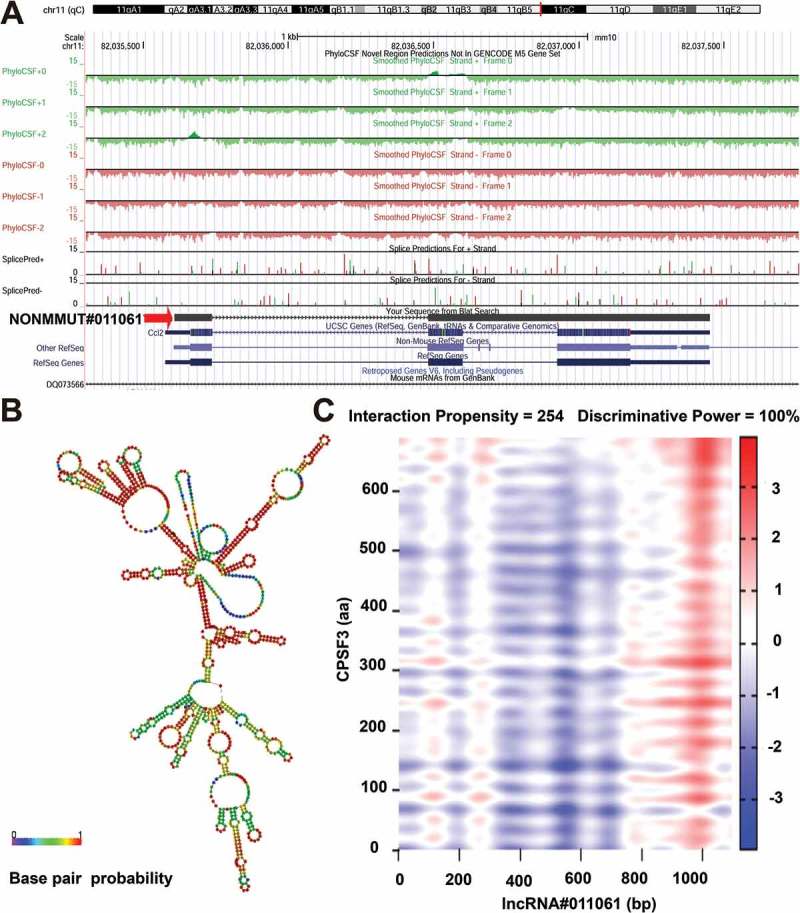
Bioinformatics analysis of NONMMUT011061. (A) The chromosome location of NONMMUT011061 in the mouse genome was shown. (B) Prediction of RNA secondary structure for NONMMUT011061 (RNAfold web server, University of Vienna). A minimal free energy structure (MFE = −396.90 kcal/mol) was shown. Base pairing probabilities have been color-coded using a scale from 0 (blue) to 1 (red). (C) catRAPID analysis indicated a strong interaction between CPSF3 and NONMMUT011061.

Since NONMMUT011061 was also distinctively stimulated during the highly pathogenic H5N1 (CK10) and H5N8 virus (QD5) infection in mice, suggesting a potential role of NONMMUT011061 in the pathogenesis of different H5 IAV. Therefore, the bio-functions of this lncRNA were further analyzed using the well-established bioinformatics tools. Transcription factors (TFs) were recognized as important components of signaling cascades controlling all types of normal cellular processes as well as response to external stimulus[47]. Here, TRANSFAC (http://www.gene-regulation.com/index2.html) was used to predict the potential TFs of NONMMUT011061. The results showed that NONMMUT011061 can combine with 74 TFs in total, including interferon regulated factor (IRF), macrophage-activating factor (MAF), heat shock factor (HSF), GATA sequence and myelocytomatosis oncogene (Myc) (Figure 8). Through University of California Santa Cruz (UCSC) Genome Browser (http://genome-asia.ucsc.edu/index.html), the genomic location of NONMMUT011061 was predicted to be on chromosome 11qB5 and was assumed to be unable to encode genes (predicated through PhyloCSF tracks: https://data.broadinstitute.org/compbio1/PhyloCSFtracks/trackHub/hub.txt) (Figure 9(A)). In silico prediction of the secondary structure of lncRNA is another useful method to define putative functions of non-coding transcripts, based on the widely-held assumption that highly-folded structures affect functionality through binding interactions with proteins/nucleotides[48]. Using RNAfold minimum free energy estimations based on RNAfold webserver (http://rna.tbi.univie.ac.at/cgi-bin/RNAWebSuite/RNAfold.cgi), a highly-folded secondary structure with several hairpin loops of NONMMUT011061 was identified (Figure 9(B)). We also conducted catRAPID analysis (http://service.tartaglialab.com) to predict the potential interacting proteins of NONMMUT011061. As a result, we found a strong interaction between NONMMUT011061 and several proteins, including cleavage and polyadenylation-specific factor 3 (CPSF3), fragile X mental retardation syndrome related protein 1 (FXR1), THO complex 1 (THOC1) and polyA-specific ribonuclease (PARN) (Figure 9(C) and Table 1).

**Table 1.** Top protein that might interact with NONMMUT011061.

Protein name	Interaction Propensity	Discriminative Power (%)
CPSF3	254	100
DHX58	244	100
NUFP2	242	100
CRNL1	228	100
THOC1	228	100
FXR1	206	100
NOP9	203	100
PARN	196	100
STRBP	184	100
CSFT	163	100

## Discussion

With the recent technical advances in genome-wide studies, it is becoming increasingly obvious that more than 98% of the human genome is transcribed into non-coding RNAs (ncRNAs), while the majority of these transcripts can be categorized as lncRNAs[49]. Although the pivotal role of individual lncRNA in the pathogenesis is being increasingly realized, the possible role of lncRNAs in the interaction between IAV and the host remains largely unknown. In this study, to further investigate the potential role of lncRNAs in the pathogenesis of IAV, a highly pathogenic virus CK10 and a low pathogenic virus GS10 were used to characterize the expression profile of lncRNAs in a mouse model using deep-sequencing. A total of 126 SDE lncRNAs were identified in CK10-infected mice, whereas the corresponding number for GS10 was only 94 (Figure 2(B)). Moreover, compared to GS10, the differentially expressed lncRNAs-coexpressed mRNAs regulated by CK10 were highly related with the aberrant and uncontrolled inflammatory responses (Figure 3–5). Interestingly, one lncRNA, NONMMUT011061, potentially interacting with a great number of inflammatory-related genes, was significantly up-regulated in both H5N1 CK10 strain and highly pathogenic H5N8 QD5 strain when compared to GS10 (Figures 3 and 9(A)).

To date, several lncRNAs have been shown to play an important role in the innate immune response against influenza virus and be associated with viral pathogenesis, including NRAV (negative regulator of antiviral response)[11], NEAT1 (nuclear enriched abundant transcript 1)[30], Bst2/BISPR (bone marrow stromal cell antigen 2 IFN-stimulated positive regulator) [31,32] and VIN (virus inducible lincRNA)[33]. NRAV inhibits the transcription of interferon-stimulated genes via affecting histone modification of these genes (mainly MxA and IFITM3), resulting in the manipulation of the antiviral response[11]. The ectopic expression of human NRAV conferred the hypersensitivity of the mice to influenza virus infection, evidenced by higher virus titers in the lung, increased body weight loss and higher mortality[11]. In addition, the cooperative action between the transcriptional regulators and nuclear lncRNAs also impacts the innate immune response to IAV infection. NEAT1, an essential lncRNA for the formation of nuclear body paraspeckles, is activated by influenza virus and involved in the transcriptional activation of the antiviral gene interleukin *Il8* probably through relocating transcriptional regulators splicing factor proline/glutamine-rich (SFPQ, a NEAT1-binding paraspeckle protein) from the I*l8* promoter to the paraspeckles[30]. In addition, lncRNAs can also directly regulate the expression of specific protein-coding-genes. LncRNA Bst2/BISPR is activated upon infection with the recombinant influenza virus that is deficient in the interferon (IFN) response blocking and after treatment with type I IFN. Bst2/BISPR regulates the expression of genomically neighboring protein-coding gene in an IFN-stimulated gene, cis bone marrow stromal cell antigen 2 (bst2), while the expression of other genes adjacent to bst2 was not affected [31,32]. It is worth noting that the host innate immune response can be regulated by the expression of NRAV, NEAT1 or Bst2/BISPR. However, VIN, a large intergenic ncRNAs, induced by H1N1, H3N2, H7N7 influenza viruses as well as vesicular stomatitis virus, plays a role in promoting influenza virus replication and is not affected by the treatment with IFN-β or IFN inducers[33].

Compared with the protein-coding sequences, the majority of lncRNAs are poorly conserved in vertebrates. Moreover, it is quite difficult to predict the functions of lncRNAs simply based on their nucleotide sequences. To reveal the functional significance of lncRNAs in IAV, we constructed the lncRNA/mRNA coexpression network based on the correlation analysis. Bio-function analysis suggested that the lncRNAs-coexpressed mRNAs induced by CK10 virus were highly associated with inﬂammatory response-related functions (Figure 4(A)). Moreover, compared to GS10 virus, mice infected with CK10 virus exhibited a stronger expression of genes associated with these functions (Figure 4(B)). Canonical pathway further demonstrated that CK10 highly activated the inflammatory cytokines-related pathways, notably, ‘Activation of IRF by Cytosolic Pattern Recognition Receptors’ and ‘Interferon Signaling’ pathways (Figure 5(A)). In addition, mice infected with CK10 exhibited a stronger expression of genes associated with these pathways (Figure 5(B-E)). Moreover, qRT-PCR results of the mRNAs that highly-related with the targeted lncRNAs forcefully confirmed that the expressions of the tested inflammatory cytokine genes were significantly higher in CK10 virus infected mouse lung than that of GS10 virus (Figure 6(C)). Thus, we surmized that the distinct expression of lncRNAs may contribute to the intense inflammatory response induced by CK10 virus which quite accordance with the histopathology observed in the mouse lung (Figure 1(G)).

Accumulating evidence suggests that a prolonged and dysregulated host response to influenza virus infection can act deleteriously to initiate or exacerbate pathological lung damage and subsequent death [50–52]. Our previous study also showed that CK10-mediated robust innate immune response, termed as a cytokine storm or hypercytokinemia, is potentially fatal and is a significant underlying factor for the high mortality of infected mice [35,36]. In this study, we found that highly-pathogenic CK10 induced higher levels of lncRNA expression than the low pathogenic strain GS10. Therefore, the large number of SDE lncRNAs induced by CK10 may play a crucial role in virus-induced hypercytokinemia. However, further studies are needed to investigate the underlying mechanism for these lncRNAs to regulate the innate immune response.

The co-expression network analysis result showed that NONMMUT011061 could correlate a number of mRNAs (Figure 3) and the qRT-PCR validation results showed that NONMMUT011061 was significantly higher expressed in CK10-infected mice than that of GS10 virus (Figure 6(A)). It is worth noting that some mRNAs highly correlated with NONMMUT011061 were also expressed at significantly higher levels compared with GS10 virus, including *Cxcl11, Cxcl5, Ccl2, Irg1, Il6, Saa1, Ccl7* and *Cxcl1* (Figure 6). In addition, NONMMUT011061 was also distinctively up-regulated by another highly pathogenic H5N8 virus in mouse lung (Figure 7(A)). Thus, these data together showed that NONMMUT011061 may act as a key regulator in IAV-mediated inflammatory response. Further functional study on NONMMUT011061 could provide useful insights into the pathogenesis of influenza virus.

Usually, the normal execution of biological event is controlled by a combination of lncRNA-mediated regulation and TFs[53]. These two mechanisms share similar regulatory logistics and cooperate in part by influencing the activity of the binding sites in target genes. Some lncRNAs, such as 7SK, can directly affect the loading and activity of general TFs and to further affect the production of mRNAs[54]. Moreover, another lncRNA, NRON, can directly serve as either co-factors or inhibitors to regulate the activity of nuclear factor of activated T cells (NFAT) proteins which manipulate gene expression in many cell types[55]. In this study, using TRANSFAC, 75 TFs were predicted to have the potential to combine with NONMMUT011061, including some TFs associated with the immune response and some important pathways, such as IRF, MAF, HSF, GATA sequence and Myc (Figure 8). Moreover, IRF, MAF, GATA and Myc are specifically up- or down-regulated during influenza virus infection (GSE41126 and GSE53932, http://www.ncbi.nlm.nih.gov/projects/geo/), suggesting their potential role during influenza virus infection.

A large number of studies have revealed the versatile and critical functions performed by IRF family in innate immunity [56–58], adaptive immunity [57] and many other biological processes, such as immune cell development [56,58], regulation of gene expression in response to pathogen infection [56,59,60],regulation of the cell cycle [61,62] and apoptosis [63,64]. The MAF is a family of transcription factor protein that belongs to the activated protein-1 super-family of transcription factors. MAF plays multiple roles in regulation of cellular development and differentiation[65]. Moreover, interaction between long intergenic non-coding RNAs (lincRNAs, linc-MAF-4) and MAF involves in the T lymphocyte differentiation[66]. In addition, an lncRNA-MAF transcription factor network plays an essential role in epidermal differentiation[67]. Interestingly, MAF also mediates the crosstalk between the MAPK and AKT/mTOR signal pathways[68]. Most importantly, a number of studies have shown that influenza virus can effectively activate the MAPK pathways and the activation of MAPK family members plays an important role in viral replication, proinflammatory and apoptotic response in various cells during influenza virus infection [69–74]. Moreover, the PI3K/Akt/mTOR pathway also activated during influenza virus infection and function as supporting viral effective replication [75–80].

HSF, a conserved stress-activated transcription factor for the heat shock proteins, is a key component of the heat shock response and plays a versatile function, such as modulating host inflammatory response[81], regulating stress-induced gene activation[82], activating the ubiquitin proteasome system to promote non-apoptotic developmental cell death[83]. The GATA is a type of transcription factor that plays important roles in several diseases, such as haematopoietic, cardiovascular, gastrointestinal tract, liver and pancreas, urogenital tract and kidney, respiratory tract, mammary gland and central nervous system diseases[84]. Moreover, the GATA also directly affects G protein signaling through regulating the expression of regulator of G protein signaling 4 (RGS4)[85]. The transcription factor Myc involves in regulating the expression of miR-23b-27b cluster during hypoxia-induced neuronal apoptosis[86]. Due to the contributions of these TFs to the regulation of the immune response and their interplay between influenza viruses, it is likely that NONMMUT011061 may serve as an important regulator of the immune response to IAV infection. Future studies are still needed to examine whether NONMMUT011061 activation is also linked to the development of autoimmune and allergic disease as well as the excessive inflammation associated with the acute lung injury caused by CK10 virus infection (Figure 1(G)).

Using the catRAPID algorithm, several proteins were predicted to be highly interactive with NONMMUT011061, including CPSF3, FXR1, THOC1 and PARN (Table 1, Figure 9(C)). CPSF3, an essential component for converting heteronuclear RNA to mRNA, is associated with cellular stress response 1 protein-mediated cell death [87] and also can be designed as an novel target for control toxoplasmosis[88]. The RNA binding protein FXR1 is a critical regulator of post-transcriptional gene expression in differentiation, development and immunity [89,90]. The THOC1, also known as hHpr1/p84, is a nuclear matrix component protein that binds to the tumor suppressor retinoblastoma protein (pRb). [91] THOC1 can dampen cell growth via inducing cell cycle arrest at G2/M and promote apoptosis in lung cancer cells and may have important implications in the development of targeted therapies for lung cancer[92]. THOC1 also plays a crucial role in embryonic development in mice [93] and regulates transcriptional elongation[94]. Deadenylation of eukaryotic mRNA is a crucial mechanism for mRNA function through affecting mRNA turnover and the efficiency of protein synthesis. PARN is one of the biochemically best-characterized deadenylases[95]. Moreover, PARN is also required for the 3′-end maturation of the telomerase RNA component (TERC) and plays a role in the biogenesis of TERC[96]. Depletion of PARN inhibits the proliferation of the gastric cancer cells and promotes cell death through arrested the gastric cancer cells at the G0/G1 phase by up-regulating the expression levels of p53 and p21[97]. Next, we want to find the potential interplay of these identified proteins with influenza virus infection. As a result, unfortunately, currently, we failed to find any association of these proteins with influenza virus infection. However, as stated above, we indeed found that these proteins have multiple functions, especially in cell death (CPSF3, THOC1 and PARN) and immunity regulation (FXR1), which might serve as a connection for the influenza virus-induced cell death and inflammatory response. Therefore, based on the multiple functions of these lncRNA-binding proteins, future investigations are warranted to explore the potential role of these proteins in IAV induced cell death and inflammatory response in the process of interacting with NONMMUT011061.

In summary, our study on the potential link between lncRNAs and IAV may presents a novel direction for understanding the pathogenesis of IAV and may give some clues of the therapeutic strategies for the disease. However, intensive studies are still needed to define the expression, regulation and functioning of lncRNAs for viral pathogenesis. Moreover, further studies centered on the common and unique lncRNAs induced by CK10 or GS10 would be helpful to further elucidate the differential pathogenic mechanisms between these two strains. In addition, further in-depth analysis of the interactions between the influenza virus machineries and specific lncRNAs (such as NONMMUT011061) will provide useful information on their potential role in IAV infection cycle. Moreover, in this study, the healthy mouse was used as the mammalian model. However, for further comparison, it is very interestingly to explore the lncRNA response using immunosuppressive, obese or pregnant mice.
