# Supplementary material for: Deep sequencing of the mouse lung transcriptome reveals distinct long non-coding RNAs expression associated with the high virulence of H5N1 avian influenza virus in mice
Source: Virulence. 2018 Jul 27;9(1):1092–111. doi: 10.1080/21505594.2018.1475795 (PMC6086314; doi:10.1080/21505594.2018.1475795)
Supplement: Supplemental Material [file kvir-09-01-1475795-s001.zip › FIG S1 GS10 Co-Expressed NetWork.docx]

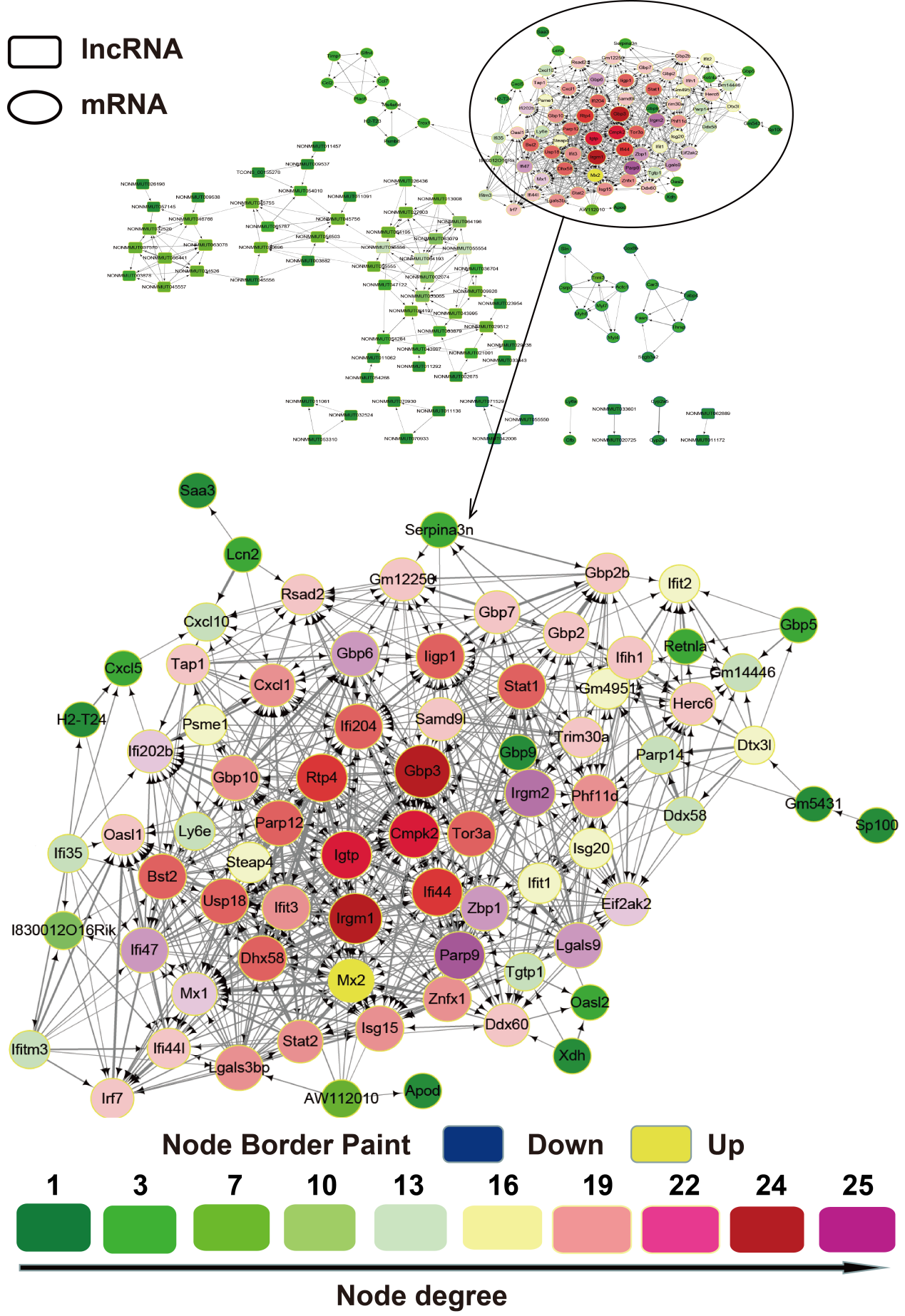


**FIG S1** The lncRNA/mRNA coexpression network constructed using the cytoscape program for the GS10 group. The lncRNAs and mRNAs with Pearson correlation coefficients ≥ 0.99 or ≤ -0.99 were selected to draw the regulatory network using the cytoscape program. In gene-coexpression networks, each gene corresponds to a node. Two genes are connected by an edge, indicating a strong correlation. Within the network analysis, a degree is the simplest, most important measure of the centrality of a gene within a network and determines the relative importance. A degree is defined as the number of directly linked neighbors. In the network, the node size indicates the node degrees and the number represents the number of directly linked neighbours that are associated with each color. Therefore, the larger the node size suggested the targeted lncRNA or mRNA could directly linked with more neighboring genes.
