# Supplementary material for: Deep sequencing of the mouse lung transcriptome reveals distinct long non-coding RNAs expression associated with the high virulence of H5N1 avian influenza virus in mice
Source: Virulence. 2018 Jul 27;9(1):1092–111. doi: 10.1080/21505594.2018.1475795 (PMC6086314; doi:10.1080/21505594.2018.1475795)
Supplement: Supplemental Material [file kvir-09-01-1475795-s001.zip › FIG S2.docx]

**
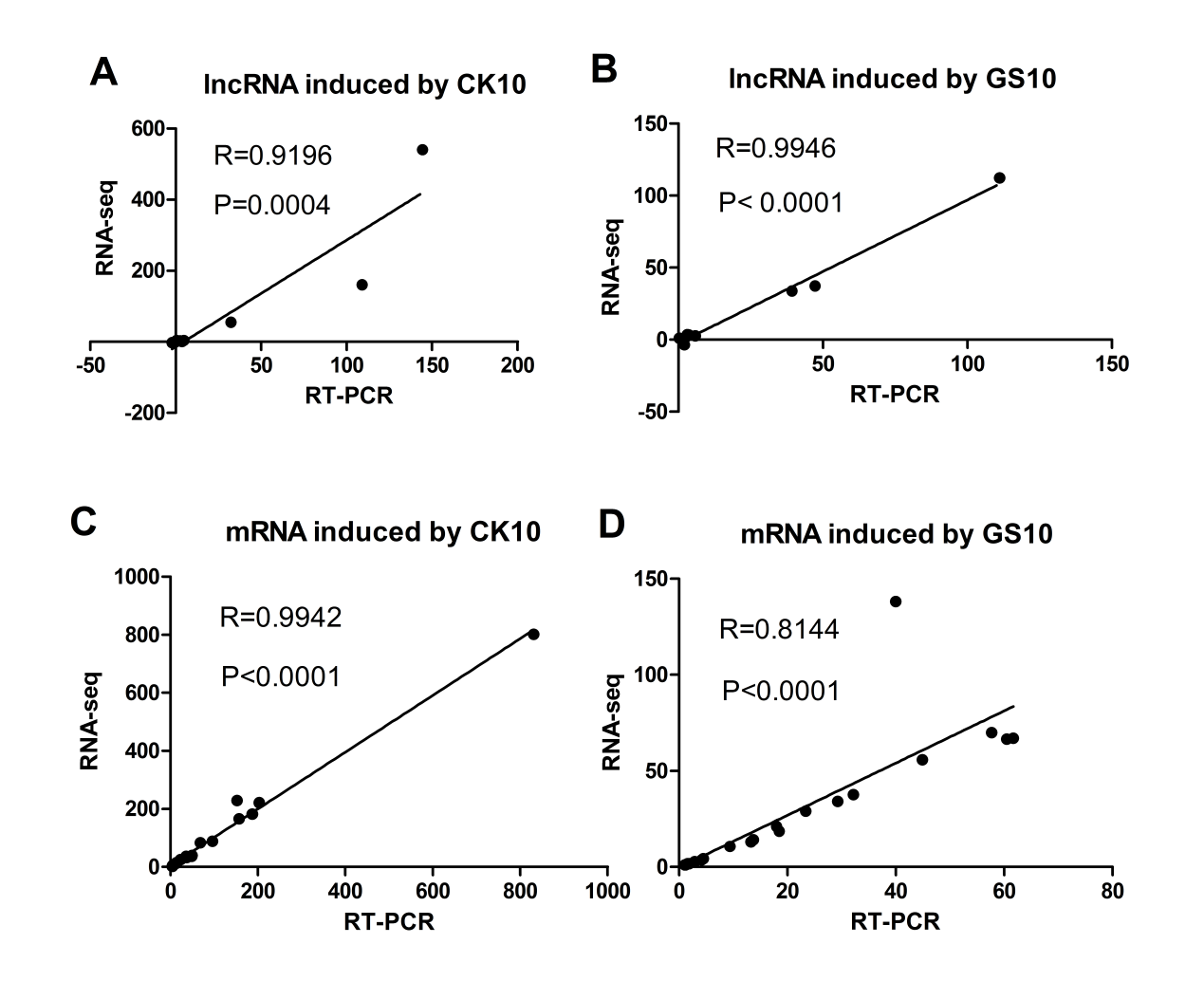
**

**FIG S2** The correlation between RNA deep-sequencing data and qRT-PCR results. The Pearson correlation test was applied to measure the significance of the correlations. (A) The correlation of lncRNA induced by CK10 between qRT-PCR and RNA-sequence data. (B) The correlation of lncRNA induced by GS10 between qRT-PCR and RNA-sequence data. (C) The correlation of mRNA induced by CK10 between qRT-PCR and RNA-sequence data. (D) The correlation of mRNA induced by GS10 between qRT-PCR and RNA-sequence data.
