# Supplementary material for: Deep sequencing of the mouse lung transcriptome reveals distinct long non-coding RNAs expression associated with the high virulence of H5N1 avian influenza virus in mice
Source: Virulence. 2018 Jul 27;9(1):1092–111. doi: 10.1080/21505594.2018.1475795 (PMC6086314; doi:10.1080/21505594.2018.1475795)
Supplement: Supplemental Material [file kvir-09-01-1475795-s001.zip › Supplementary information.docx]

**>NONMMUT011061**

CACCAGCACCAGCCAACTCTCACTGAAGCCAGCTCTCTCTTCCTCCACCACCATGCAGGTCCCTGTCATGCTTCTGGGCCTGCTGTTCACAGTTGCCGGCTGGAGCATCCACGTGTTGGCTCAGCCAGATGCAGTTAACGCCCCACTCACCTGCTGCTACTCATTCACCAGCAAGATGATCCCAATGAGTAGGCTGGAGAGCTACAAGAGGATCACCAGCAGCAGGTGTCCCAAAGAAGCTGTAGTGTGAGTTACATACCCCGGCCCTCCCTGGTCCAAAGGTTTTTCCTTAAGAACAAGGGATGGTCCTCATATACTTATAGTCAGTCACACACTCAGATCCAATGGGGAAACCAAGGCCAAGAAGGCAAAGGCAGTTCTCAACAGCATTGTCTCTATGGCTGCTGTTCAGGCCCTTTCTACTCCACAAGCTTATCTTAGAAAACCTGCAGGAGAAGCAGGTCACTTTGAGTCCCCTTTTTCTACCTGCCCTCCCCCGCTGAGCTCTACACAGCCCCTCCATGTATACCAGACTGAACTTCATCTAACAGTGTCTTTTCTCTTCCCACAGTTTTGTCACCAAGCTCAAGAGAGAGGTCTGTGCTGACCCCAAGAAGGAATGGGTCCAGACATACATTAAAAACCTGGATCGGAACCAAATGAGATCAGAACCTACAACTTTATTTAAAACTGCATCTGCCCTAAGGTCTTCAGCACCTTTGAATGTGAAGTTGACCCGTAAATCTGAAGCTAATGCATCCACTACCTTTTCCACAACCACCTCAAGCACTTCTGTAGGAGTGACCAGTGTGACAGTGAACTAGTGTGACTCGGACTGTGATGCCTTAATTAATATTAAACTTATTTAACTTATTGATGTTATGGTATTCCCTTTCATGAATACTAAAATTTCTTAAATGCAAGGTGTGGATCCATTTTTCCCTCTCTGTGAATCCAGATTCAACACTTTCAATGTATGAGAGATGAATTTTGTAAAGATGAATGGGTAAACTTTGTGTTTGAGATTCCAAGGTATTGTTTAAAATATTATTATGGATATTCCttattattaaaagaaatatattatttttGTACA
